# Supplementary material for: Estimating the Effect of School Water, Sanitation, and Hygiene Improvements on Pupil Health Outcomes
Source: Epidemiology. 2016 Aug 2;27(5):752–60. doi: 10.1097/EDE.0000000000000522 (PMC4969057; doi:10.1097/EDE.0000000000000522)
Supplement: Supplementary file 1 [file ede-27-752-s001.docx]

**ABSTRACT**

**Background:** We conducted a cluster-randomized water, sanitation, and hygiene trial in 185 schools in Nyanza province, Kenya. The trial, however, had imperfect school-level adherence at many schools. The primary goal of this study was to estimate the causal effects of school-level adherence to interventions on pupil diarrhea and soil-transmitted helminth infection.

**Methods:** Schools were divided into water availability groups, which were then randomized separately into either water, sanitation, and hygiene intervention arms or a control arm. School-level adherence to the intervention was defined by the number of intervention components—water, latrines, soap—that had been adequately implemented. The outcomes of interest were pupil diarrhea and soil-transmitted helminth infection. We used a weighted generalized structural nested model to calculate prevalence ratios (PR).

**Results:** In the water-scarce group, there was evidence of a reduced prevalence of diarrhea among pupils attending schools that adhered to two or to three intervention components (PR = 0.28, 95% CI: 0.10, 0.75), compared to what the prevalence would have been had the same schools instead adhered to zero components or one. In the water available group, there was no evidence of reduced diarrhea with better adherence. For the soil-transmitted helminth infection and intensity outcomes, we often observed point estimates in the preventive direction with increasing intervention adherence, but primarily among girls, and the confidence intervals were often very wide.

**Conclusions:** Our instrumental variable (IV) point estimates sometimes suggested protective effects with increased water, sanitation, and hygiene intervention adherence, although many of the estimates were imprecise.

## INTRODUCTION

In spite of biological plausibility and a long-standing history of epidemiologic studies supporting the preventive effects of water, sanitation, and hygiene on health (e.g. John Snow),^1-4^ the results from rigorous school-based water, sanitation, and hygiene trials have been mixed.^5-7^ For example, we conducted a cluster-randomized water, sanitation, and hygiene trial in 185 schools in Nyanza province, Kenya (2007–2009), and the intention-to-treat results showed reduced diarrhea,^7^ but only among the most water scarce schools that also received a water provision; and reduced soil-transmitted helminth infection, but primarily among girls and primarily for the *Ascaris lumbricoides* worm, but not other soil-transmitted helminths.^6^ However, there was imperfect school-level adherence at many schools, which may have contributed to these trial results.

Most trials report the intention-to-treat effect—the average causal effect of randomization on the outcome—regardless of adherence to the intervention.^8^ However, if adherence to the intervention was poor, the intention-to-treat results may be very different from what would have been observed under conditions of good adherence. In our trial, because adherence to the intervention was imperfect, the intention-to-treat estimates may be a distorted measure of the causal effect of water, sanitation, and hygiene intervention itself, and may merit supplementation with a valid causal effect measuring adherence on the outcome.^9,10^ The public health significance of understanding the effects of adherence to water, sanitation, and hygiene interventions is that if one could see that negative or mixed trial results were due to imperfect adherence, then it might lead researchers to focus simply on improving adherence rather than on finding alternative water, sanitation, and hygiene technologies.

There are several methodologies that might estimate the effect of actual adherence—as opposed to the effect of randomization—on outcomes. Commonly used approaches such as the ‘as-treated’ and ‘per-protocol’ analyses are likely to suffer from unmeasured confounding.^11^ IV analyses use the randomized assignment variable as an instrument to control for confounding. There are a number of IV frameworks, although some present difficulties in estimation for complex trial designs.^12^ The principal stratification framework is easily interpretable based on potential outcomes, but in trials with multiple intervention arms it becomes increasingly complex to identify the principal strata.^12^ For example, a three-armed trial (like ours) would have 27 principal strata, and estimation of parameters within each principal stratum requires complex modeling assumptions. The endogenous regressor framework^13^ is often used, but can be problematic for binary and count outcomes, like ours.^12^ The structural nested model framework, which was first developed by Robins^14^ and has since been generalized by others,^12,15,16^ provides a robust framework allowing it to be used in complex trial designs like ours, which has multiple intervention arms, cluster-level randomization, complex sampling schemes, and a variety of outcome types.

We performed an IV analysis to estimate the causal effects of school-level adherence to water, sanitation, and hygiene interventions on several pupil-level health outcomes, including pupil diarrhea, pupil soil-transmitted helminth infection, and soil-transmitted helminth infection intensity. We hypothesized that the preventive effects of water, sanitation, and hygiene would be more pronounced among schools with better adherence to the intervention.

## METHODS

Our data are from a cluster-randomized trial that was designed to assess the impact of school-based water, sanitation, and hygiene interventions on various health and educational outcomes, where the intention-to-treat results for these outcomes have already been reported.^6,7,17-19^ The IV results for the diarrhea and soil-transmitted helminth outcomes have not been previously reported, and are the emphasis of this study. The study took place between 2007 and 2009 in 185 rural primary schools in what were formerly four districts of Nyanza Province, Kenya—Rachuonyo, Suba, Nyando, and Kisumu.

*School selection and randomization:* All selection criteria were determined in collaboration with implementing partners and the Kenyan Government. An initial survey was sent out to all primary schools in the geographic area (n=1,084) to assess water, sanitation, and hygiene conditions; 83% of the surveys were returned. Schools in certain administrative divisions and schools with pupil to latrine ratios that already met the Government of Kenya standard (25:1 for girls and 30:1 for boys) were ineligible for the study, leaving 289 eligible schools.^20^ Schools were then divided into two groups based on whether or not they had an improved water source within one kilometer during the dry season, and 135 ‘water available’ schools and 50 ‘water scarce’ schools were randomly allocated into study arms using a random number generator, with the allocation stratified by geographic district.

The randomization processes in the two water availability study groups were separate. In the water available group, 135 schools were randomly allocated into *three* study arms of equal size: 1) the water available control arm; 2) the hygiene promotion and water treatment arm; and 3) the hygiene promotion and water treatment, plus sanitation improvement arm. In the water scarce group, 50 schools were randomly allocated into two study arms of equal size: 1) the water scarce control arm; and 2) the hygiene promotion and water treatment, plus sanitation, plus water supply improvement arm. The soil-transmitted helminth study was nested within the larger trial, and took place among a randomly selected subset of 39 schools from Rachuonyo and Nyando/Kisumu that were already taking part in the water available group of the larger study.

All control schools received the interventions at the end of the study. Further details on the interventions are available elsewhere.^18^ It was not possible to mask schools, data collectors, or pupils to the intervention arm to which the school had been randomized.

*Pupil selection:* For both the diarrhea and soil-transmitted helminth outcomes, a random sampling scheme was used to select pupils from the school registers, sampling by sex and grade. For the diarrhea outcome, at each of the 185 schools, 25 pupils from grades 4–8 were randomly selected to participate in the study. For the soil-transmitted helminth outcomes, at each of the 39 primary schools, 25 pupils, from grades 3-5 were randomly selected (distinct from the selection that took place in the diarrhea study). Different children were sampled at the baseline and follow-up visits.

*Data collection:* Data were collected by trained enumerators from the Great Lakes University of Kisumu. Enumerators visited the schools unannounced on a randomly selected weekday within the study period. School WaSH characteristics were collected both by direct observation and by structured interviews with head teachers in the English language. Pupils were interviewed about their water, sanitation, and hygiene knowledge, attitudes and practices, and about self-reported health and educational outcomes in the Dholuo language. Baseline data collection took place between February and March of 2007. After randomization and implementation, data for the first follow-up were collected between April and October of 2008.

*Outcomes:* For the diarrhea study, our outcome of interest was pupil-reported diarrhea (binary), defined as three or more loose or watery stools over any 24–hour period in the previous week.^21^ In the diarrhea study, we did not have any missing data due to non-response or refusal to participate.

For the soil-transmitted helminth study, all sampled pupils provided stool specimens that were analyzed for *Ascaris lumbricoides*, *Trichuris trichiura*, and any species of hookworm using the Kato-Katz method.^22,23^ The outcomes of interest were soil-transmitted helminth infection (binary), and secondarily the intensity of infection measured in eggs per gram of feces, both of which were assessed by individual helminth species. All children attending any of the 185 schools received yearly deworming (400mg of albendazole). In the helminth study, 22 observations were deleted due to non-response.

*Adherence:* We measured adherence to three separate school-level water, sanitation, and hygiene intervention components on the day of the study visit: 1) soap availability, 2) safe water availability, and 3) latrine acceptability.^12^ Soap availability was defined as the school having handwashing soap near the latrines for pupil use. Safe water availability was defined as the school either having available water from an improved water source, or having available water with detectable chlorine from any source. Latrine acceptability was based on the school having an adequate number of latrines that were maintained and structurally intact.

In an ‘as-treated’ analysis, one could compare all eight possible combinations of the above intervention components. For our IV analysis, we needed to reduce the dimensionality of the adherence variable. First, not all of the possible combinations existed, and second, IV analyses only allow as many effect estimates as instruments.^12^ We defined adherence by the number of water, sanitation, and hygiene intervention components to which each school adhered. We first created a composite, 4-level variable that is the sum of whether or not there was soap available (yes=1, no=0), safe water available (yes=1, no=0), and acceptable latrines at the school (acceptable=1, not acceptable=0). This four-level adherence variable can be used as either a continuous variable or can further be categorized. In the water scarce schools and in the helminth study, we only had one instrument, and we present results both using the continuous adherence variable, and also the dichotomous variable (i.e. adherence to > 2 components vs. < 2). In the water available schools, we had two instruments due to having three randomization arms, so we were able to produce two adherence estimates using a single model (i.e. adherence to 1-2 components vs. 0, and adherence to 3 components vs. 0).

*Safety and confidentiality:* Institutional Review Board approval was obtained from The Emory University Institutional Review Board (Atlanta, GA, USA). Permission to conduct the trial was also granted by the Government of Kenya Ministries of Health, Water and Irrigation, and Education. Oral assent was obtained from all participants, and approval was also obtained by head teachers of each school in *loco parentis*.

*Analysis*

*Weights:* Because this trial was cluster randomized, individual-level balance of covariates is not guaranteed by randomization, so we also produced weights (W_ij1_) that were used to remove the association between individual-level confounders and randomization (eAppendix 1; http://links.lww.com/EDE/B70).^12^ Diarrhea and soil-transmitted helminths have different mechanisms of infection, and so we collected and controlled for different individual variables in the two studies. Diarrhea is transmitted through a fecal-oral pathway, and for this outcome we used weights to control for pupils’ age. Because the helminths under study are soil-transmitted, for these outcomes we used weights to control for pupils’ age, and additionally for pupils’ shoe-wearing behavior (important for hookworm), and for geophagy (a soil-eating practice). Sampling weights were also produced to account for the unequal probability of selection of individuals into the study. These weights (W_ij2_) are the inverse of the probability of selection of each pupil into the study. We call the product of these two weights W_ij_, which is an overall weight that accounts simultaneously for the complex sampling of pupils and for confounding by individual covariates (eAppendix 1; http://links.lww.com/EDE/B70).^12^

*Structural nested models:* We used a weighted generalized structural nested mean model:^12^

*h*(E^W1^(Y_ij_(a)|A_i_=a, R_i_)) - *h*(E^W1^(Y_ij_(0)|A_i_=a, R_i_)) = a_v_ξ.

Y_ij_(a) represents the potential outcome for the j^th^ pupil in the i^th^ school at some observed adherence level a, A_i_ represents either a categorical or continuous school-level adherence variable, R_i_ represents a categorical school-level randomization variable, E^W1^ represents a weighted expectation (e.g. using the weight W_ij1_), *h* represents a link function (e.g. *h*(p) = p; *h*(p) = log(p); *h*(p) = log(p/(1-p))), and ξ represents a causal effect—for example a RD, logRR, or logOR corresponding to the link function that was used to transform the left parts of the model. The subscript v (e.g. a_v_) denotes a vector function, perhaps when there are several levels of categorical adherence. The structural nested model framework is based on potential outcomes, and conditions on observed adherence—the effect of actually receiving treatment. Note that only Y_ij_(a) is actually observed, whereas the potential outcome Y_ij_(0) is a counterfactual that is modeled. Potential outcomes under no treatment are observed only in the control and non-adherent clusters, and not observed in adherent clusters.

To solve for the structural nested model’s causal parameter (i.e. ξ), first, the independence assumption is employed to construct unbiased estimating equations—i.e. equations with mean zero. These estimating equations are then solved using Newton-Raphson—a method that uses Taylor series approximation, and an initial guess of ξ and iteratively finds an approximation of ξ. Our method differs from G-estimation, in that G-estimation solves a different estimating equation, and often using a grid search method to do so. The mathematical details of these estimating equations and the iterative algorithm to solve them are summarized in eAppendix 1(http://links.lww.com/EDE/B70), eAppendix 2(http://links.lww.com/EDE/B71) and elsewhere.^12^

Researchers using structural nested models often only target the parameter ξ, which is conditional on both A and R, but of particular interest to us, was to calculate a PR that was conditional only on A. Specifically, the effects of interest for the diarrhea outcome and soil-transmitted helminth infection outcomes were the PRs comparing the prevalence of disease among adherers to what the prevalence of disease would have been had this same group not adhered: PR(a)= (E^W1^(Y_ij_(a)|A_i_=a)) / E^W1^(Y_ij_(0)|A_i_=a)). This effect is known as the effect of the treatment on the treated in less complex settings. The numerator of interest from the PR, i.e. E^W1^(Y_ij_(a)|A_i_=a), is easily calculated without the structural nested model. This can be done, for example, by regressing Y_ij_ on A_i_ in SAS version 9.4 (Cary, NC, USA) using PROC REG (while using the W_ij_ weight) and outputting the ‘parameter estimates’ for each participant. The denominator, E^W1^(Y_ij_(0)|A_i_=a), is a counterfactual and is calculated from the structural nested model parameters. Rather than the causal parameter (i.e. ξ), of particular interest from the structural nested model is h(E^W1^(Y_ij_(0)|A_i_=a, R_i,_), which differs from our IV denominator of interest in that it is also conditional on R and also that a link function is applied to it. We can apply the inverse link function (e.g. the expit, or exponential function) to h(E^W1^(Y_ij_(0)|A_i_=a, R_i_) to produce a counterfactual prevalence of disease for each participant in the study. To then make these conditional prevalences marginal on only A, we regress E^W1^(Y_ij_(0)|A_i_, R_i_) on A_i_, again using PROC REG (while using the W_ij_ weight) and outputting the parameter estimates. The resulting prevalences, E^W1^(Y_ij_(0)|A_i_=a), represent the true potential outcome had a participant’s school counterfactually not adhered to the intervention (e.g. had it been assigned to R=0). The IV parameter of interest (a PR) is this numerator divided by this denominator.

The PR can be produced for the diarrhea and soil-transmitted helminth infection outcomes (both binary) with either the log, logistic, or linear structural nested model.^12^ We experimented with all three structural nested models, but chose to use the logistic one as it had the best fit—it was the only one that always produced a solution for each of our relationships of interest. For the soil-transmitted helminth intensity of infection outcomes, which are counts, the IV parameters are calculated similarly, except that log-linear SNM is used, to produce a ratio comparing the average EPG among pupils in adhering schools to what the EPG count would have been had these same schools not adhered.

We used, and make available, SAS programs for the log, logistic, and linear SNMs, which were originally created by Brumback et al. (see eAppendix 1; http://links.lww.com/EDE/B70).^12^ These SAS programs were used to estimate the PR and 95% confidence intervals, with only minimal modifications to the original program. Our study was a cluster randomized trial, where we handled clustering of the individual level confounders using weights (i.e. W_ij1_), and we handled clustering in the estimation of the variance by assuming schools were independent and jackknifing schools. These SAS programs could also be modified and used for individual-level trials (or possibly even observational studies that meet the study assumptions), by modifying the weights, and by jackknifing individuals instead of clusters.

*Structural Nested Model Assumptions:* The validity of the estimates, including the ability of the structural nested model to account for unknown/unmeasured school-level confounding, is dependent upon meeting a number of study assumptions that are described below, and also described in greater detail elsewhere.^12^

1) We assume the exclusion restriction is met, which is that there are no direct effects of randomization assignment on the outcome (see directed acyclic graph in Figure 1). This assumption requires that randomization of a school to receive water, sanitation, and hygiene interventions does not directly prevent (or cause) diarrhea/soil-transmitted hygiene infection except through adherence to the intervention.

2) We make the consistency assumption, which is that the observed outcomes are potential outcomes under the treatment/adherence level that was actually received/observed.^24^ In our specific study, this means that when we observe school-level adherence at a given level (e.g. a=3), that this observed adherence is intrinsically linked to a well-defined potential outcome.

3) The structural nested model's effects are based on potential outcomes, with the assumption that potential outcomes are independent of the randomization variable, or in a cluster randomized trial like ours, that the potential outcomes are independent of the randomization variable, conditional on individual-level baseline covariates. We used weights, as discussed earlier, to remove the association between individual-level confounders and randomization. In the Figure 1 directed acyclic graph, this weighting would remove the arrow from the individual-level confounders to randomization.

4) We assume the distribution of potential outcomes in our data satisfy a structural nested model. Implied in the structural nested model is a no interaction assumption, that ξ, the model’s causal effect, is the same within the different randomization groups. If the prevalence or mean of a covariate at a given adherence level is imbalanced across randomization groups, then this assumption requires that the covariate is not an effect modifier of the causal effect of adherence on the outcome. If there are unknown effect modifiers, then this assumption may not be met. However, if effect modifiers are known, the assumption can still be satisfied by stratifying upon them. For example, sex was reported to be an effect modifier of the water, sanitation, and hygiene intervention on soil-transmitted helminth infections^6^ so to better meet this assumption, we stratify all the helminth analyses by sex.

We carried out our analyses in SAS version 9.4 (Cary, NC, USA). We make available SAS programs for the log, logistic, and linear structural nested models (eAppendix 1; http://links.lww.com/EDE/B70), which were obtained from Brumback et al.^12^ and modified to allow for variations in our study. The jackknife variance estimation procedure^25^ was also built into the SAS program and used to estimate 95% confidence intervals. Further details on both parameter estimation and variance estimation are summarized in eAppendix 2 (http://links.lww.com/EDE/B71) and elsewhere.^12^

*Intention-to-treat analyses:* We compared the intention-to-treat and IV estimates. ORs (not PRs) for the diarrhea,^7^ and soil-transmitted helminth infection^6^ outcomes had been previously reported. We recalculated all the intention-to-treat estimates using the overall weight (W_ij_) to assure that a fair comparison was being made between our intention-to-treat and IV analyses. We used the following model to estimate the PR for the three-armed trial:

$$\log\left( \mu_{\mathrm{ij}} \right)=\alpha_{0}+\beta_{1}{Intervention1}_{i}+\beta_{2}{Intervention2}_{i}$$

$\mu_{\mathrm{ij}}$ is the expectation of the response variable—either binary diarrhea, binary soil-transmitted helminth infection, or a count of helminth eggs per gram of feces. The school-level water, sanitation, and hygiene interventions are represented by dummy variables corresponding to each of the two intervention arms, with the control arm as the referent. In the two armed trial, only $\beta_{1}$ would be included in the model. The coefficients and 95% confidence intervals were produced using survey procedures in SAS-Callable SUDAAN version 11.0.1. (Research Triangle Park) and reflect disproportionate sampling of pupils within schools, the clustering of pupils within schools, and the stratified randomization by geographical districts.

## RESULTS

*Baseline characteristics:* Baseline descriptive statistics, by randomization arm, are shown in Table 1. We generally had balance of school-level and individual-level covariates between the intervention arms.

*Water, sanitation, and hygiene intervention adherence:* School-level adherence to the interventions at the first follow-up is shown in Table 2. In each of the three study groups, we observed that the schools allocated into the intervention arms adhered to more intervention components than schools allocated into the control arms. Although we observed an increase in the level of adherence in all intervention arms across the study, we also observed that only 29 schools fully adhered to all three water, sanitation, and hygiene components.

*Diarrhea:* Using the categorical adherence variable, we observed that increased adherence to two or more intervention components was associated with a reduced prevalence of diarrhea (PR = 0.28, 95% CI: 0.10, 0.75; Table 3). When using the continuous adherence variable, pupils attending water-scarce schools that adhered to two of the components had a reduced prevalence of diarrhea prevalence compared to what the diarrhea prevalence would have been had the same schools not adhered to any intervention components (PR = 0.27, 95% CI: 0.11, 0.69; Table 3). The contrast comparing perfectly adhering schools to non-adhering schools (using the same ξ from the structural nested model above using a continuous adherence variable, but for a three-unit change) revealed a strong but extremely imprecise estimate (PR = 0.094, 95% CI: 0.0043, 2.1). For comparison, the intention-to-treat effect, which compares all water-scarce intervention schools to all water scarce control schools without regard to intervention adherence, was PR=0.38 (95% CI: 0.20, 0.73).

In the water available schools, the PR for pupils in schools that adhered to all three water, sanitation, and hygiene components was 0.71 (95% CI: 0.22, 2.4; Table 3), and the PR for pupils in schools adhering to either one or two components was (1.9, 95% CI: 0.12, 29). Both effects had wide confidence intervals.

*Soil-transmitted helminth infection:* All of the IV point estimates among girls were in the preventive direction (eTable 1; http://links.lww.com/EDE/B72), and the point estimates for *A. lumbricoides* (PR=0.23, 95% CI: 0.045, 1.2) and hookworm (PR=0.26, 95% CI: 0.055, 1.2) were particularly strong, although imprecise. For boys, the hookworm IV point estimate was also in the preventive direction (PR=0.25, 95% CI: 0.41, 1.5), but the *A. lumbricoides* and *T. trichiura* point estimates were both slightly above the null.

*Soil-transmitted helminth infection intensity:* The direction and strength of the PRs for the soil-transmitted helminth intensity outcomes often mirrored the pattern that we observed for the helminth infection outcomes, although the confidence intervals were always much wider for the helminth intensity outcomes (eTable 2; http://links.lww.com/EDE/B73).

## DISCUSSION

Our study used an instrumental variable analysis to estimate the effect of actual water, sanitation, and hygiene intervention adherence—as opposed to the effect of randomization—on pupil diarrhea and pupil soil-transmitted helminth infection. Our IV results from the water scarce schools suggested a strong preventive effect of adherence to interventions on reducing pupils’ diarrhea, whereas results were less clear in water available schools and in the soil-transmitted helminth subset of schools.

Imperfect adherence may be one reason why this and other previous school water, sanitation, and hygiene trials have not provided strong evidence of the efficacy of school-based programs for the prevention of diarrheal disease and soil-transmitted helminth infections.^5-7^ The strong preventive point estimates that we often observed with increasing adherence lend to the theory that adherence may be an important factor in previous trials’ mixed results. However, the confidence intervals were often wide, and in the presence of such imprecision, stronger estimates may actually contain less information.^26^ It was encouraging that with the ‘continuous adherence IV model,’ both the IV prevalence ratio *and its upper bound* were further from the null than the intention-to-treat model prevalence ratio and its upper bound, respectively (Adherence to 2 components vs. 0 components from Table 3).

The null IV results for several of the outcomes is suggestive that there are also probably a number of other factors, besides adherence, that are important in reducing these infectious diseases. It is not clear why our results were so different in water scarce versus water available schools. One possibility is that the water, sanitation, and hygiene interventions in the water scarce schools were more comprehensive, notably including a *community-level* water source. It may be that this access to water was a final sufficient component that allowed individuals to practice water, sanitation, and hygiene activities both at school, and possibly also at home and elsewhere in the community. It also may be that the water source intervention simply provided better access to microbiologically safe water.

We made several efforts to best meet the study assumptions, including using the structural nested model with the best fit, stratifying upon known effect modifiers, and thoughtfully specifying individual-level confounders. We do believe that the IV study assumptions could be plausibly met, however, some assumptions are untestable. Further methods development might be merited to evaluate these assumptions in this complex trial settings.

Our study is not without limitations. Our inability to blind water, sanitation, and hygiene interventions and our need to reduce the dimensionality of adherence due to only having two instruments could impair our ability to meet the exclusion restriction assumption. Further, the continuous adherence structure necessitates an assumption of a linear relationship between adherence and the outcome on either the logit or log scale. There is also the possibility of unknown individual-level confounders, or unknown effect modifiers that we may not have accounted for. Finally, stratification by sex in the soil-transmitted helminth study, possibly exaggerates the problem of wide confidence intervals that is already inherent to many IV analyses.^27,28^

# REFERENCES

1. Wagner EG, Lanoix JN. Excreta Disposal for Rural Areas and Small Communities. *WHO Monograph series*. Vol. No 39. Geneva: WHO, 1958.

2. Ferriman A. BMJ readers choose the “sanitary revolution” as greatest medical advance since 1840. *BMJ* 2007;**334**(7585):111.

3. Fewtrell L, Kaufmann RB, Kay D, Enanoria W, Haller L, Colford JM, Jr. Water, sanitation, and hygiene interventions to reduce diarrhoea in less developed countries: a systematic review and meta-analysis. *Lancet Infect Dis* 2005;**5**(1):42-52.

4. Clasen TF, Bostoen K, Schmidt WP, Boisson S, Fung IC, Jenkins MW, Scott B, Sugden S, Cairncross S. Interventions to improve disposal of human excreta for preventing diarrhoea. *Cochrane Database Syst Rev* 2010(6):CD007180.

5. Jasper C, Le TT, Bartram J. Water and sanitation in schools: a systematic review of the health and educational outcomes. *Int J Environ Res Public Health* 2012;**9**(8):2772-87.

6. Freeman MC, Clasen T, Brooker SJ, Akoko DO, Rheingans R. The impact of a school-based hygiene, water quality and sanitation intervention on soil-transmitted helminth reinfection: a cluster-randomized trial. *Am J Trop Med Hyg* 2013;**89**(5):875-83.

7. Freeman MC, Clasen T, Dreibelbis R, Saboori S, Greene LE, Brumback B, Muga R, Rheingans R. The impact of a school-based water supply and treatment, hygiene, and sanitation programme on pupil diarrhoea: a cluster-randomized trial. *Epidemiol Infect* 2014;**142**(2):340-51.

8. Hernan MA, Robins JM. Instruments for causal inference: an epidemiologist's dream? *Epidemiology* 2006;**17**(4):360-72.

9. Greenland S, Lanes S, Jara M. Estimating effects from randomized trials with discontinuations: the need for intent-to-treat design and G-estimation. *Clin Trials* 2008;**5**(1):5-13.

10. Matsuyama Y. A comparison of the results of intent-to-treat, per-protocol, and g-estimation in the presence of non-random treatment changes in a time-to-event non-inferiority trial. *Stat Med* 2010;**29**(20):2107-16.

11. Little RJ, Rubin DB. Causal effects in clinical and epidemiological studies via potential outcomes: concepts and analytical approaches. *Annu Rev Public Health* 2000;**21**:121-45.

12. Brumback BA, He Z, Prasad M, Freeman MC, Rheingans R. Using structural-nested models to estimate the effect of cluster-level adherence on individual-level outcomes with a three-armed cluster-randomized trial. *Stat Med* 2014;**33**(9):1490-502.

13. Wooldridge JM. *Econometric Analysis of Cross Sectional and Panel Data*. Cambridge, MA: The MIT Press:, 2002.

14. Robins JM. Correcting for Noncompliance in Randomized Trials Using Structural Nested Mean Models. *Communications in Statistics-Theory and Methods* 1994;**23**(8):2379-2412.

15. Korhonen PA, Laird NM, Palmgren J. Correcting for non-compliance in randomized trials: an application to the ATBC Study. *Stat Med* 1999;**18**(21):2879-97.

16. Vansteelandt S, Goetghebeur E. Causal inference with generalized structural mean models. *Journal of the Royal Statistical Society: Series B* 2003;**65**(4):817–835.

17. Dreibelbis R, Freeman MC, Greene LE, Saboori S, Rheingans R. The impact of school water, sanitation, and hygiene interventions on the health of younger siblings of pupils: a cluster-randomized trial in Kenya. *Am J Public Health* 2014;**104**(1):e91-7.

18. Freeman MC, Greene LE, Dreibelbis R, Saboori S, Muga R, Brumback B, Rheingans R. Assessing the impact of a school-based water treatment, hygiene and sanitation programme on pupil absence in Nyanza Province, Kenya: a cluster-randomized trial. *Trop Med Int Health* 2012;**17**(3):380-391.

19. Garn JV, Greene LE, Dreibelbis R, Saboori S, Rheingans RD, Freeman MC. A cluster-randomized trial assessing the impact of school water, sanitation, and hygiene improvements on pupil enrollment and gender parity in enrollment. *J Water Sanit Hyg Dev* 2013;**3**(4).

20. Republic of Kenya Ministry of Education. MOE National School Water, Sanitation, and Hygiene Promotion Strategy: 2008–2015 Nairobi, 2008.

21. Baqui AH, Black RE, Yunus M, Hoque AR, Chowdhury HR, Sack RB. Methodological issues in diarrhoeal diseases epidemiology: definition of diarrhoeal episodes. *Int J Epidemiol* 1991;**20**(4):1057-63.

22. Katz N, Chaves A, Pellegrino J. A simple device for quantitative stool thick-smear technique in Schistosomiasis mansoni. *Rev Inst Med Trop Sao Paulo* 1972;**14**(6):397-400.

23. Basic laboratory methods in medical parasitology. Geneva: World Health Organization, 1991.

24. Cole SR, Frangakis CE. The consistency statement in causal inference: a definition or an assumption? *Epidemiology* 2009;**20**(1):3-5.

25. Korn EL, Graubard BI. *Analysis of Health Surveys*. New York: John Wiley & Sons, 1999.

26. Morris CN. Comment. *JASA* 1987;**82**(397):131-133.

27. Davies NM, Smith GD, Windmeijer F, Martin RM. Issues in the reporting and conduct of instrumental variable studies: a systematic review. *Epidemiology* 2013;**24**(3):363-9.

28. Swanson SA, Hernan MA. Commentary: how to report instrumental variable analyses (suggestions welcome). *Epidemiology* 2013;**24**(3):370-4.

**Figure legends**

**Figure 1.** Directed acyclic graph (DAG) describing study.

**List of Supplemental Digital Content**

**eAppendix 1.** Structural nested model SAS programs. pdf

**eAppendix 2.** Structural nested model estimation. pdf

**eTable 1.** ITT and IV prevalence ratios for school WaSH adherence and pupil STH infection. docx

**eTable 2.** ITT and IV prevalence ratios for school WaSH adherence on pupil STH intensity in eggs per gram (EPG) of feces. docx
